# Versatility of 14-3-3 proteins and their roles in bone and joint-related diseases

**DOI:** 10.1038/s41413-024-00370-4

**Published:** 2024-10-15

**Authors:** Renpeng Zhou, Weirong Hu, Peter X. Ma, Chuan-ju Liu

**Affiliations:** 1 Department of Orthopaedics and Rehabilitation, Yale University School of Medicine, New Haven, CT USA; 2 Department of Biologic and Materials Sciences and Prosthodontics, School of Dentistry, University of Michigan, Ann Arbor, MI USA

**Keywords:** Bone, Diseases

## Abstract

Bone and joint-related diseases, including osteoarthritis (OA), rheumatoid arthritis (RA), and bone tumors, pose significant health challenges due to their debilitating effects on the musculoskeletal system. 14-3-3 proteins, a family of conserved regulatory molecules, play a critical role in the pathology of these diseases. This review discusses the intricate structure and multifunctionality of 14-3-3 proteins, their regulation of signaling pathways, and their interactions with other proteins. We underscore the significance of 14-3-3 proteins in the regulation of osteoblasts, osteoclasts, chondrocytes, and bone remodeling, all key factors in the maintenance and dysfunction of bone and joint systems. Specific focus is directed toward elucidating the contribution of 14-3-3 proteins in the pathology of OA, RA, and bone malignancies, where dysregulated 14-3-3-mediated signaling cascades have been implicated in the disease processes. This review illuminates how the perturbation of 14-3-3 protein interactions can lead to the pathological manifestations observed in these disorders, including joint destruction and osteolytic activity. We highlight cutting-edge research that positions 14-3-3 proteins as potential biomarkers for disease progression and as innovative therapeutic targets, offering new avenues for disease intervention and management.

## Introduction

Bone and joint-related diseases, such as osteoarthritis (OA), rheumatoid arthritis (RA), gout, osteoporosis, and various bone tumors, represent a significant burden on global health due to their widespread prevalence and the pain and disability they cause.^1,2^ These conditions arise from a complex interplay of genetic predispositions, metabolic dysregulations, inflammatory responses, and biomechanical stresses, culminating in the gradual degradation of joint integrity and bone structure.^3–6^ The impact of these diseases is profound, not only in terms of patient quality of life but also in economic terms, due to the costs associated with long-term management and treatment.^7^ Therefore, it is essential to prioritize the search for a highly effective therapeutic target.

The 14-3-3 protein family, discovered in bovine brain homogenate in 1967, comprises a group of highly conserved and homologous proteins that are ubiquitously expressed in all eukaryotic cells.^8^ These proteins were initially identified by Tony Moore and colleagues based on their fractionation pattern in DEAE-cellulose chromatography and starch-gel electrophoresis. Since then, 14-3-3 proteins have been renamed multiple times (e.g., BAP-1, Bilardo, Exo1) due to their involvement in various regulatory processes.^9^ Structurally, 14-3-3 proteins consist of nine α-helices forming an amphipathic groove, which facilitates their interaction with a multitude of binding partners. These proteins are encoded by different genes, resulting in multiple isoforms, such as β, γ, ε, η, σ, τ/θ, and ζ in mammals, each encoded by distinct genes yet sharing a fundamental structural framework and displaying propensity for both homo- and heterodimeric formations.^10,11^ The 14-3-3 proteins, found in diverse cellular compartments such as the cytoplasm, plasma membrane, nucleus, and mitochondria,^12^ participate in a wide range of cellular processes, such as signal transduction, cell cycle regulation, apoptosis, and neurodevelopment, underscoring their significance in maintaining cellular homeostasis and their potential as diagnostic and therapeutic targets across various diseases.^13–15^

The 14-3-3 proteins have been implicated in various biological processes, acting as molecular scaffolds that stabilize protein conformations, prevent degradation, and influence the activity and subcellular localization of their binding partners.^14,16^ Regulatory mechanisms of 14-3-3 proteins are predominantly centered around their ability to bind to specific phosphoserine/phosphothreonine-containing motifs in target proteins, thereby modulating their function and localization.^17,18^ 14-3-3 proteins are primarily regulated through phosphorylation, as most targets must be phosphorylated to bind to 14-3-3.^19^ This interaction is crucial for the regulation of various signaling pathways and cellular functions such as apoptosis, cell cycle progression, and neurodevelopment. With over 1200 identified binding partners, the 14-3-3 family serves as a central node in numerous signaling pathways,^20,21^ affecting a wide range of diseases and making them a significant focus for drug discovery and therapeutic intervention.

Within the realm of bone and joint-related diseases, growing evidence implicates 14-3-3 proteins in the regulation of critical pathways governing bone metabolism and joint health. For instance, 14-3-3 proteins emerge as significant modulators playing roles in osteoblast, osteoclast, and chondrocyte differentiation, as well as in bone remodeling.^22–25^ Moreover, the involvement of 14-3-3 proteins in inflammatory pathways suggests a potential role in inflammatory joint diseases such as RA.^26^ The regulatory functions of 14-3-3 proteins in apoptosis and cell cycle control also point to their importance in the pathophysiology of bone tumors and osteoporosis.^27,28^ Consequently, 14-3-3 proteins emerge as potential molecular diagnostic markers and therapeutic targets in bone-related diseases.^29–31^ Therefore, the following section will delve into the mechanistic regulation of 14-3-3 proteins, assess their distinct roles in bone- and joint-related diseases, and introduce innovative perspectives for treating these conditions.

## Structure of 14-3-3 proteins

**Fig. 1:**
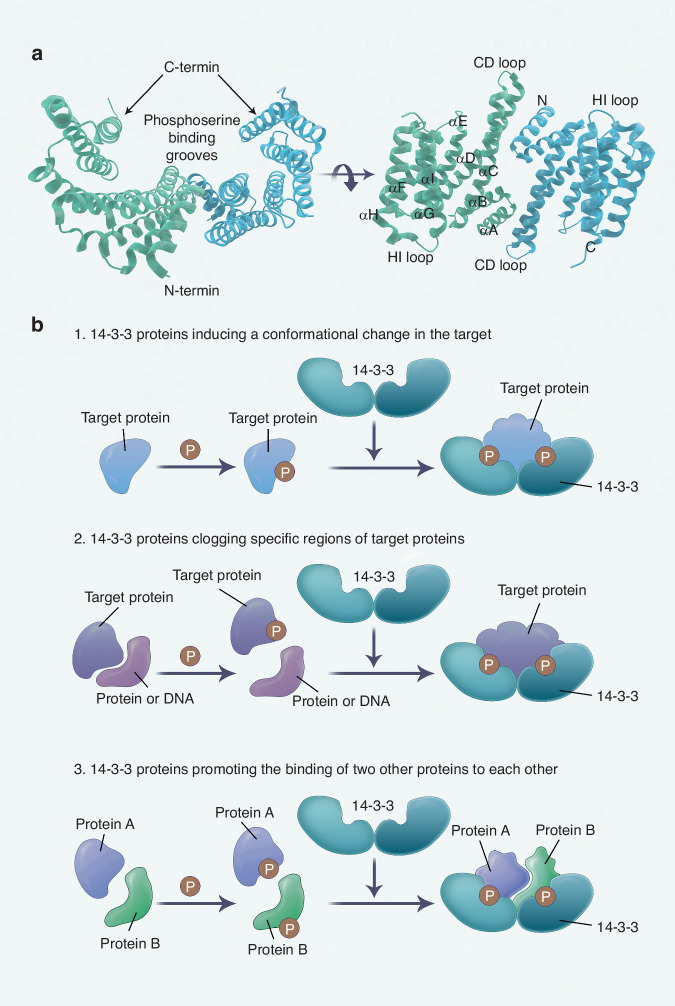
Model for 14-3-3 structure and function. **a** Structure of the 14-3-3 dimer. Each monomer has a different color. The helices of a monomer are labeled αA-αI. The N-terminus, C-terminus, and specific loops (CD and HI) are labeled to depict the overall topology of the protein. Helices and loops involved in target domain interactions are labeled, highlighting the phosphoserine binding grooves that facilitate interaction with phosphorylated target proteins. **b** Modes of 14-3-3 protein action. (1) 14-3-3 proteins induce a conformational change in the target protein, recruiting target proteins to the groove of the 14-3-3 dimeric structure and modulating enzymatic activity; (2) 14-3-3 proteins clog specific regions of target proteins, i) interfering with protein-protein or protein-DNA interactions, ii) regulating the subcellular localization of target proteins, iii) preventing protein dephosphorylation or degradation; or (3) 14-3-3 proteins promote the binding of two other proteins to each other, with the two target proteins immobilized in the 14-3-3 dimer groove, promoting their interaction

The molecular mass of 14-3-3 proteins is ~30 kD, and they predominantly exist as dimers. The dimer forms a “flattened horseshoe” shape, creating a central channel that measures 35 Å broad, 35 Å wide, and 20 Å deep.^10^ Each monomer subunit is composed of nine α-helices arranged into four sections: two pairs of helices (αA-αB, αC-αD), another pair (αE-αF), and a trio of helices (αG-αI). The first four α-helices are essential for dimerization, as they form the dimer interface and a sizeable aperture at the subunit interface.^32^ This configuration allows helices αA and αB of one monomer to interact with helices αC and αD of the other, stabilizing the dimer structure. The conserved peptide-binding groove is formed by helices αC, αE, αG, and αI, featuring a positively charged patch on one side and a hydrophobic patch on the other. The central channel’s inner wall is characterized by invariant residues at the dimer interface, while variable residues appear on the outer convex surface.^33^ Two ligand-binding grooves are present within the channel, including a phosphorylation-binding groove (PBG) comprised of a cluster of helical αE-H residues. PBG selectively binds phosphorylated serine or threonine residues, facilitating 14-3-3’s function. Mutations in PBG can impair various cellular functions typically mediated by 14-3-3.^34,35^ Despite the C-terminal extension’s apparent lack of functional significance,^35^ the N-terminal’s short amphiphilic helices are crucial for membrane interactions and protein binding (Fig. 1).

Seven isoforms of 14-3-3, named with Greek letters (β, ε, η, γ, τ, σ, ζ), are coded by distinct genes (*YWHAB*, *YWHAE*, *YWHAH*, *YWHAG*, *YWHAQ*, *SFN*, *YWHAZ*, respectively). These isoforms are conserved but have varying tendencies to form homo- or heterodimers, leading to significant structural variations.^16,33^ The ε isoform, for example, predominantly forms heterodimers, rarely homodimers.^32^ Isoform-specific differences also exist in salt bridge structures. All isoforms share the Arg18-Glu89 salt bridge, but some, like ε, η, γ, and σ, lack the Glu5-Lys74 bridge, and ε lacks the third, Asp21-Lys85.^10^ The C-terminal extensions vary among isoforms and are generally disordered.^18^

## Function of 14-3-3 proteins

14-3-3 proteins play a crucial role in cellular signaling, with a wide range of functions. 14-3-3 proteins, as conserved entities, involve in various biological processes, including mitotic signaling, cell cycle progression, metabolism, tumorigenesis, and apoptosis, by interacting with diverse client proteins.^36^ These interactions occur in three principal ways: inducing conformational changes in target proteins to fit within the 14-3-3 dimer groove, sequestering target proteins to interfere with their interactions, and facilitating binding between proteins within the dimer groove^11,33^ (Fig. 1).

## Modification functions of 14-3-3 proteins

### Stabilization of protein structure and activity

The 14-3-3 proteins form a highly conserved family crucial to stabilizing protein structures and modulating their activities, thereby acting as key regulators within numerous signaling pathways. These proteins bind to an extensive range of phosphorylated client proteins, significantly influencing their stability and function.^37^ 14-3-3 proteins specifically recognize and associate with motifs containing phosphoserine or phosphothreonine.^38^ This binding, facilitated by the 14-3-3 dimer’s central channel, often induces a conformational change that stabilizes the client protein in a functional state.^18^ For instance, 14-3-3 proteins promote cell survival by binding to phosphorylated BAD protein, sequestering it from the mitochondrial membrane and preventing apoptosis.^39^ 14-3-3 proteins also bind to tau fragments with phosphorylated residues pSer214 and pSer324 and contribute to its aggregation.^40^ Furthermore, 14-3-3 proteins maintain the structural and functional stability of a variety of kinases.^41^ They play a dual regulatory role in the activation of Raf-1 kinase within the ERK-MAPK pathway, keeping it inactive in the absence of extracellular signals and stabilizing its active configuration when extracellular signals are present.^13^ Their broad interaction with kinases emphasizes their essential role in signal transduction.^18^ In addition, 14-3-3 proteins serve as scaffolds, promoting the assembly of protein complexes that enhance the specificity and efficiency of signaling pathways. This scaffolding stabilizes multiprotein assemblies, such as those in the large G protein-coupled receptor (GPCR) signaling complexes.^42^ Evidence indicates that 14-3-3 proteins serve as sorting factors, directing GPCR recycling and trafficking. Recent studies show that GPCRs differentially and temporally engage with various 14-3-3 isoforms, likely in distinct subcellular locations.^43^

### Regulation of ligand nuclear translocation

The binding of 14-3-3 proteins to client proteins plays a crucial role in the regulation of nuclear translocation of transcription factors. By modulating nuclear localization sequences (NLS), 14-3-3 proteins can regulate ligand subcellular localization.^44^ For instance, caspase-2, which possesses an NLS and requires nuclear localization for its activation preceding cell death, is hindered in its nuclear translocation when bound to phosphorylated 14-3-3, thus preventing its activation.^40,41,45^ Similarly, Notch receptors, which are key determinants of cell fate, are restrained from entering the nucleus by 14-3-3 interactions, thereby controlling gene transcription.^46^ The JAK-STAT signaling pathway demonstrates the promoting or inhibiting roles of 14-3-3 in nuclear translocation. Activation of cytokine membrane receptors, such as interleukin-6 (IL-6), initiates JAK kinase to phosphorylate STAT tyrosine residues, facilitating the nuclear translocation of STAT and the regulation of gene transcription.^47,48^ However, the interaction of 14-3-3 proteins with JAK1 phosphorylated at Ser515 or Ser518 modulates STAT nuclear translocation; simultaneous phosphorylation at both sites inhibits binding to 14-3-3 and is believed to attenuate JAK-STAT signaling, thereby diminishing STAT-mediated transcription.^37^ Conversely, the association of 14-3-3ζ with STAT3 at Ser727 can promote this signaling pathway’s activity, whereas the reduction of 14-3-3ζ is associated with decreased phosphorylation at this site and reduced pathway activity.^37^

Additionally, 14-3-3 proteins protect critical phosphorylation sites from dephosphorylation, thus preserving the active or inactive states of client proteins as required for proper cellular function.^49^ This is evident in the regulation of the forkhead box O (FOXO) family of transcription factors, where the binding of 14-3-3ζ prevents dephosphorylation and nuclear translocation of FOXO proteins, thereby influencing the transcription of genes involved in cell cycle arrest and apoptosis.^46,47^ Furthermore, 14-3-3 proteins maintain the phosphorylation status of non-binding motif sites, such as those inhibiting the activity of calcium/calmodulin-dependent protein kinases 1 and 2, thereby playing a crucial role in cellular regulation by affecting the structure and accessibility of kinase domains and autoinhibitory segments.^18^ 14-3-3 proteins also promote tau phosphorylation.^50,51^ In this manner, 14-3-3 proteins are not only pivotal in ligand nuclear translocation but also in maintaining the phosphorylation status crucial for the regulation of protein activity, highlighting their comprehensive role in cellular signaling pathways.

## Biological functions

### Cell cycle

14-3-3 proteins promote cell survival by antagonizing proapoptotic proteins.^52^ The cell cycle, comprising synthesis (S) and mitotic (M) phases, with gap phases (G1 and G2), is critical for cell proliferation and fate. 14-3-3 proteins regulate the cell cycle by interacting with CDC25 phosphatases, CHK1, and Wee1 kinase, which are key regulators coordinating cell cycle activation and repression.^53^ It has been described that 14-3-3 protein can delay S phase entry by binding to CDC25A at Ser178 and Thr507.^53^ 14-3-3 also recruits to E2F7/8 gene promoters, disrupting transcription and preventing cell cycle arrest.^54^ Specific isoforms, like 14-3-3β, induce G2/M phase arrest, whereas 14-3-3τ can inhibit cell proliferation via the IL6/JAK2/PI3K pathway.^55^ Through these interactions, 14-3-3 proteins precisely regulate the cell cycle, ensuring accurate cell division and proper cellular responses to both internal and external signals.

### Apoptosis

14-3-3’s role in apoptosis is well-established. It regulates apoptosis by binding to the pro-apoptotic protein BAD, a Bcl-2 family member. Phosphorylation of BAD at Ser111, Ser112, and Ser136 by p21-Activated Kinase 1 is associated with apoptosis regulation.^56^ Additionally, phosphorylation at S118 using phosphomimetic (S118D) mutants of BAD enhances S99 phosphorylation, which promotes 14-3-3 binding and cell survival.^39^ Isoforms like 14-3-3ε and 14-3-3θ/τ inhibit apoptosis by suppressing apoptosis signal-regulating kinase 1 (ASK1) activation.^57^ Conversely, 14-3-3ζ‘s role in promoting β-catenin’s nuclear translocation demonstrates its anti-apoptotic effects.^58^ Interestingly, overexpression of 14-3-3γ can promote apoptosis by reducing phosphorylation of key signaling molecules.^59^ Overall, 14-3-3 proteins regulate apoptosis by interacting with crucial regulatory proteins and pathways, thereby affecting cell fate decisions.

### Autophagy

Autophagy, a cellular stress regulator, includes chaperone-mediated autophagy, microautophagy, and macroautophagy.^60^ 14-3-3ζ, by binding to Beclin 1’s S295 site, prevents its degradation, enhancing autophagy through VPS34 binding.^61^ The PI3K/AKT/mTOR pathway, crucial for autophagy, is modulated by 14-3-3’s interaction with IRS1.^53^ Crotonylation of 14-3-3ε releases PPM1B, activating autophagy by dephosphorylating ULK1.^62^ New research on lipid autophagy, where 14-3-3 synergizes with various proteins to degrade lipid droplets, highlights its role in maintaining lipid metabolism.^63^ Collectively, these findings highlight the important role of 14-3-3 proteins in regulating autophagy, providing potential avenues for therapeutic intervention.

### Metabolism

Metabolic regulation is vital for energy balance and cell growth. 14-3-3 proteins are involved in the regulation of cellular metabolism, including amino acid metabolism and energy homeostasis.^64^ 14-3-3ζ interacts with metabolic enzymes, influencing metabolic pathways.^65^ It regulates AMPK’s localization and activity, which is crucial for sensing changes in cellular energy and nutrient status.^66,67^ KAT2A-induced *YWHAZ* expression by H3K79 succinylation illustrates 14-3-3ζ‘s role in promoting glycolysis.^68^ 14-3-3σ‘s inhibitory effect on metabolic processes like glycolysis and mitochondrial biogenesis has also been noted.^69^ These diverse roles of 14-3-3 proteins highlight their central importance in maintaining metabolic balance and cellular function.

## 14-3-3 protein interactions in cellular signaling pathways

**Fig. 2:**
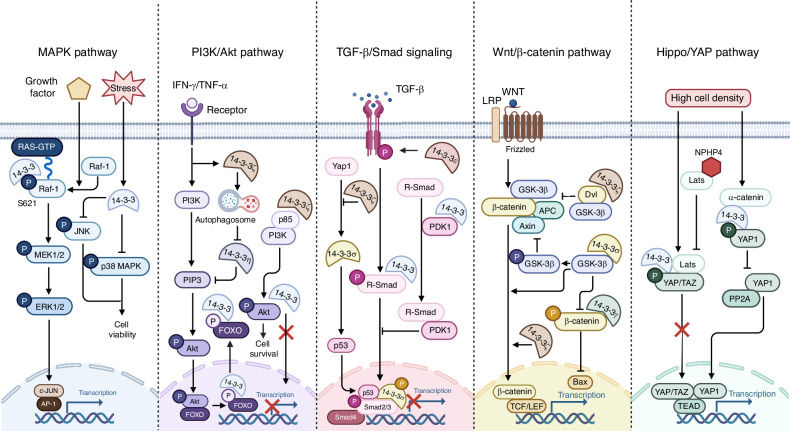
Roles of 14–3-3 proteins in MAPK, PI3K/AKT, TGF-β/Smad, Wnt/β-Catenin and Hippo/YAP signaling pathway. In the MAPK pathway, 14-3-3 regulates the activation of ERK1/2, JNK, and p38, influencing the transcriptional activity of c-JUN and AP-1, and modulating cell viability. In the PI3K/AKT pathway, 14-3-3 interacts with p85, AKT, and FOXO, affecting the activity of transcription factors like FOXO and regulating cell cycle and proliferation. In the TGF-β/Smad pathway, 14-3-3 interacts with Smad and PDK1, among others, influencing downstream transcription expression and participating in functional regulation. Within the Wnt/β-Catenin pathway, 14-3-3 interacts with β-Catenin, Dvl, and GSK-3β, thereby impacting downstream transcription expression and contributing to the regulation of cell apoptosis. Lastly, in the Hippo/YAP pathway, 14-3-3 interacts with phosphorylated Yap/TAZ or YAP1, influencing downstream transcription expression and participating in the regulation of cell proliferation. Figure created with BioRender.com

**Fig. 3:**
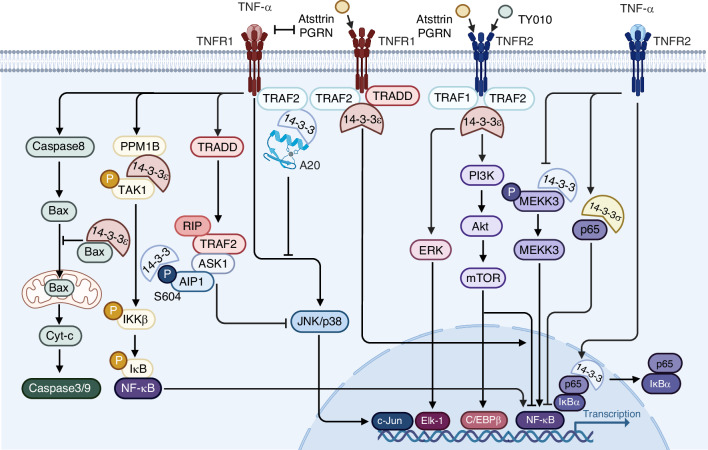
The role of 14-3-3 proteins in the TNFR signaling pathway. Interacting with key signaling mediators downstream of TNFR activation, 14-3-3 proteins regulate apoptosis, inflammation, cell survival, and proliferation at various junctures. Upon TNF-α stimulation, both TNFR1 and TNFR2 trigger a multitude of intracellular signaling cascades. Notably, 14-3-3 directly associates with the TNFR2 receptor complex, potentially modulating the effects of progranulin (PGRN) and Atsttrin, which directly bind to TNFR2 and influence its signaling outcomes. This interaction may have implications for the regulation of downstream signaling events such as the activation of the PI3K/Akt pathway, ERK, and NF-κB transcriptional activity, all of which are critical for cell survival, proliferation, and immune responses. Furthermore, the binding of 14-3-3 to phosphorylated MEKK3 and p65 within the TNFR2 signaling branch suggests a role in fine-tuning the balance between pro-survival and pro-inflammatory signals. Figure created with BioRender.com

The 14-3-3 proteins are a family of regulatory molecules that play a critical role in cellular signaling by interacting with a diverse set of client proteins. These interactions are typically phosphorylation-dependent, with 14-3-3 proteins recognizing specific phosphoserine- or phosphothreonine-containing motifs. Through their binding, 14-3-3 proteins can alter the conformation, localization, and function of their target proteins, which consequently modulates various signaling pathways, including MAPK, PI3K/AKT, TGF-β/Smad, Wnt/β-Catenin, Hippo/YAP, TNFR Signaling Pathway (Figs. 2 and 3).

### MAPK pathway

14-3-3 proteins act as molecular scaffolds to facilitate the activation of the Ras/MAPK cascade. Upon growth factor stimulation, Raf-1 becomes phosphorylated at specific serine residues, generating binding sites for 14-3-3. The binding of 14-3-3 dimers to phosphorylated Raf-1 induces a conformational change that relieves the autoinhibited state of Raf-1, promoting its interaction with Ras-GTP at the plasma membrane.^70,71^ This membrane recruitment and activation of Raf-1 initiates the sequential phosphorylation and activation of the downstream kinases MEK and ERK, ultimately leading to the regulation of transcription factors that control gene expression involved in cell proliferation, differentiation, and survival.^72^ In stress response, 14-3-3 proteins bind to phosphorylated JNK and p38 MAPK, affecting their activity and the cellular response to stressors.^73,74^

### PI3K/Akt pathway

The PI3K/Akt pathway is a critical signaling cascade involved in cell survival, metabolism, and growth. 14-3-3 proteins interact with and regulate several components of this pathway. In the PI3K/Akt pathway, 14-3-3 proteins interact with multiple nodes to fine-tune signaling outputs. Upon activation of the pathway, AKT is phosphorylated and subsequently binds to 14-3-3 proteins, stabilizing its active conformation and preventing dephosphorylation-induced inactivation.^75,76^ Furthermore, 14-3-3 proteins can directly bind to phosphorylated Akt, promoting its cytoplasmic localization and enhancing its kinase activity towards specific substrates.^75,77^ Additionally, 14-3-3 proteins interact with other components of the PI3K/AKT pathway, such as p85α kinases, further modulating pathway activity. 14-3-3ζ isoform can promote cancer cell survival by binding to the p85α regulatory subunit of PI3K and activating Akt.^78,79^ Downstream of Akt, 14-3-3 proteins bind to and sequester FOXO transcription factors in the cytoplasm, inhibiting their pro-apoptotic and cell cycle arrest functions.^77,80^

### TGF-β/Smad signaling

The TGF-β/Smad signaling pathway plays a pivotal role in regulating cellular processes such as proliferation, differentiation, and epithelial-mesenchymal transition (EMT). 14-3-3 proteins interact with and modulate the activity of Smad proteins, which are the key signal transducers in this pathway. Upon TGF-β receptor activation, the receptor-regulated Smads, such as Smad2 and Smad3, become phosphorylated at their C-terminal SSXS motif, creating a binding site for 14-3-3 proteins.^81^ The formation of this Smad/14-3-3 complex facilitates the nuclear translocation of the Smads by masking their nuclear export signal.^82^ Once in the nucleus, the Smad complexes associate with other transcriptional co-activators and co-repressors to regulate the expression of target genes involved in the processes like cell growth, differentiation, and EMT.^83^ Specifically, 14-3-3 can sequester phosphorylated Smad3 in the cytoplasm, thereby inhibiting its transcriptional activity and the expression of TGF-beta-responsive genes.^82^ This effect of 14-3-3 serves as a regulatory checkpoint, ensuring that Smad-mediated transcription is tightly controlled and occurs in response to appropriate TGF-beta signaling cues.

### Wnt/β-catenin pathway

The Wnt/β-catenin signaling pathway is crucial for embryonic development and tissue homeostasis in adults. Central to this pathway is the regulation of β-catenin, whose stability and localization are modulated by 14-3-3 proteins. These proteins have dual roles: they can prevent the degradation of β-catenin in the canonical Wnt pathway, promoting gene transcription essential for cell fate and proliferation, and they can influence cell polarity and movement via the Disheveled protein in the non-canonical pathway.^84^ Without Wnt signaling, 14-3-3 proteins bind to β-catenin, which is then phosphorylated by the destruction complex, leading to ubiquitination and degradation.^58,85^ However, when Wnt is present, it inhibits the destruction complex, resulting in β-catenin accumulation and nuclear translocation. Additionally, 14-3-3 proteins have been shown to inhibit GSK-3β activity, enhancing β-catenin stabilization.^86^ Once inside the nucleus, β-catenin partners with TCF/LEF transcription factors to initiate the transcription of Wnt-responsive genes, which govern cell proliferation and stem cell maintenance.^87,88^

### Hippo/YAP pathway

The 14-3-3 proteins are instrumental in the regulation of the Hippo signaling pathway, a critical determinant of cellular proliferation and apoptosis, which in turn influences organ size and tumor suppression.^89^ Within the canonical Hippo pathway, a kinase cascade phosphorylates the transcriptional coactivators YAP and TAZ at specific sites that are recognized by 14-3-3 proteins. The binding of 14-3-3 to these phosphorylated sites on YAP/TAZ leads to their cytoplasmic sequestration, effectively inactivating their pro-proliferative functions by preventing their interaction with TEAD transcription factors. This regulatory mechanism is essential for maintaining normal skin homeostasis and is modulated by additional proteins as elucidated by Schlegelmilch et al.^90^ They discovered that α-catenin, a component of cell-cell junctions, acts as a cell density sensor and stabilizes the YAP1/14-3-3 complex by protecting YAP1 from dephosphorylation by PP2A, thereby preventing YAP1’s nuclear entry. Furthermore, NPHP4 has been identified as a negative regulator of 14-3-3 binding, interacting with Lats1 kinase to inhibit the phosphorylation of TAZ/YAP, leading to their nuclear accumulation and a pro-proliferative effect, which has implications for the pathogenesis of nephrophthisis.^91^ These findings underscore the complexity of 14-3-3’s role in the Hippo pathway and its impact on cell growth and disease.

### TNFR signaling pathway

The 14-3-3 protein family, particularly the epsilon isomer, plays a pivotal regulatory role in the TNF signaling pathway, which is integral to inflammatory responses and disease pathogenesis. 14-3-3ε modulates the TNF-α-induced activation of NF-κB, a key transcription factor in inflammation and immune responses.^26^ By dynamically interacting with components of the MAPK signal module, such as TAK1 and PPM1B, 14-3-3ε selectively influences the time course-dependent NF-κB activity.^92,93^ This interaction is crucial for the fine-tuning of inflammatory responses, preventing excessive cytokine production that can lead to pathologies like cancer. Furthermore, 14-3-3 proteins exhibit a binding partner of the TNFR receptor, particularly TNFR2, facilitating its activation and subsequent downstream signaling pathways implicated in anti-inflammatory responses and tissue protection. Progranulin (PGRN), a multifaced growth factor-like molecule,^94–99^ and its derivative Atsttrin,^100–105^ plays a pivotal role in regulating TNFR signaling, particularly through its interaction with TNFR1 and TNFR2. Acting as a decoy receptor ligand, PGRN competes with TNF-α for binding to TNFR1, effectively dampening TNF-α-induced inflammatory signaling and mitigating pro-inflammatory pathways.^6^ Additionally, PGRN directly binds to TNFR2, triggering its signaling pathway and promoting anti-inflammatory effects and tissue protection in various disease models, including OA.^106^ In the context of OA, 14-3-3ε is a component of the TNFR2 receptor complex in chondrocytes, where its activation by PGRN and Atsttrin, exerts a protective effect, signaling through ERK-dependent pathways while suppressing NF-κB.^26,29^ In addition, 14-3-3 proteins are essential for the nuclear export of p65-IκBα complexes, terminating NF-κB signaling and maintaining cellular homeostasis.^107^ 14-3-3σ, another isomer, acts as a tumor suppressor in breast cancer by regulating the nuclear export of p65-NF-κB, affecting cancer cell migration and metastasis.^108^ The regulatory functions of 14-3-3 proteins in the TNFR signaling pathway, suggesting their potential as therapeutic targets and prognostic indicators for various inflammation-related diseases.

## Modulators of 14-3‑3 protein−protein interactions

The 14-3-3 family of proteins, recognized as central hubs in numerous cellular processes, have emerged as significant targets for pharmacological intervention, particularly in the context of diseases such as cancer, neurodegeneration, and musculoskeletal disorders. Modulators of 14-3-3 protein-protein interactions (PPIs) are broadly classified into two categories: inhibitors, which disrupt the 14-3-3/client protein interactions, and stabilizers, which enhance these interactions.^20^ The structural basis for the modulation of 14-3-3 PPIs has been elucidated through X-ray crystallography, revealing the amphipathic groove of 14-3-3 proteins as the primary site for ligand binding. This structural characterization has been instrumental in guiding the design of both inhibitors and stabilizers, leveraging structure-based drug design and computational tools such as SeeSAR for optimization.^11,109^ The modulation of these interactions presents a promising therapeutic avenue, especially in contexts where dysregulation of these pathways is evident. The development of small molecule modulators, which can either inhibit or stabilize 14-3-3 PPIs, has been a focus of recent research efforts, aiming to harness the therapeutic potential of targeting these interactions.

Inhibitors of 14-3-3 PPIs, such as the R18 peptide and its dimeric form difopein, operate by competitively binding to the conserved groove of 14-3-3 proteins, thus preventing the association with phosphorylated client proteins. The R18 peptide, identified through phage display, contains a central WLDLE motif that mimics the phosphoserine contact points, allowing it to bind without the need for phosphorylation. This interaction effectively inhibits the binding of proteins like Raf-1, ASK1, and exoenzyme S to 14-3-3, modulating downstream signaling pathways. Difopein, with its two R18 units linked by a flexible spacer, can bind to one or two monomers of 14-3-3, offering a potent competitive inhibition mechanism. Small molecule inhibitors such as BV02 have also been identified, which disrupt the 14-3-3/c-Abl interaction, leading to the release of c-Abl and its pro-apoptotic functions, suggesting potential therapeutic applications in chronic myeloid leukemia.^110,111^

Stabilizers of 14-3-3 PPIs, on the other hand, are designed to enhance the interaction between 14-3-3 proteins and their client proteins. This approach is exemplified by compounds such as pyrrolidone1 and epibestatin, which were discovered through biochemical screening and structural analysis. These stabilizers mimic the action of natural products like fusicoccin A by binding to the 14-3-3/client protein complex, thereby reinforcing the interaction and potentially increasing the functional expression of the client proteins. The design of stabilizers is inherently complex, as these molecules must bind simultaneously to 14-3-3 and its partner protein. However, the specificity mediated by the compound’s simultaneous binding to the highly variable rim of the PPI interface offers a unique opportunity for targeted manipulation of the extensive 14-3-3 interactome.^111,112^

The development of 14-3-3 modulators, both inhibitors and stabilizers, requires a deep understanding of the structural and functional nuances of 14-3-3 interactions. The availability of cocrystal structures of 14-3-3 isoforms in complex with interaction partners has been instrumental in guiding structure-based optimization of these modulators. This has opened up possibilities for the refinement of existing compounds to achieve higher affinity and specificity. Furthermore, the specificity and selectivity of these modulators for particular 14-3-3 isoforms or client proteins are critical considerations, as the development of isoform—or client-specific compounds could lead to more targeted and effective therapeutic strategies. The use of computational methods such as high-throughput docking and pharmacophore screening has been successful in identifying novel modulators, highlighting the potential of these tools in the discovery and optimization of 14-3-3 PPI modulators.^109,110^

The modulation of 14-3-3 PPIs represents a promising therapeutic strategy, with inhibitors and stabilizers offering distinct mechanisms of action to regulate these interactions. The continued refinement of these modulators, guided by structural insights and computational analyses, holds the potential to yield highly specific and effective therapeutic agents. As the field advances, it is anticipated that additional molecules will be published, furthering the exciting field of 14-3-3 PPI modulation and its implications in chemical biology and drug development (Table 1).^11,112^

**Table 1:** Stabilizers and inhibitors of 14-3-3 protein-protein interactions (PPI)

Category	Name	Structure/formula	IC_50_/EC_50_	Function	Host cell	Refs
14-3-3 PPI Stabilizers	Fusicoccin A		EC_50_ = 29 mmol/L	Enhances the 14-3-3/H^+^ATPase interaction, leading to constitutive activation of the H^+^ATPase and the pathogenic effect	Ba/F3	^111^
	Cotylenin A		/	Stabilizes 14-3-3C-RAF interactions	Epidermoid and rectal carcinoma	^227^
	Fusicoccin THF		EC_50_ = 12.4 μmol/L	Result in a 20-fold stabilization of the interaction between 14-3-3 and the potassium channel TASK3	Xenopus oocytes transfected to express human TASK3	^228^
	ISIR-005		/	Stabilizes the cancer-relevant interaction of the adaptor protein 14-3-3 and Gab2	Human cancer cells	^229^
	Pyrrolidone1		EC_50_ = 101 μmol/L	14-3-3/PMA2 stabilizers, pyrrolidone1 interacted in a solvent accessible site comparable to that of FCA	/	^230^
	Epibestatin		/	14-3-3/PMA2 stabilizers, mimic the fusicoccin effect by stabilizing the 14-3-3/client protein interaction	/	^230^
	Pyrazole34		EC_50_ = 383 μmol/L	14-3-3/PMA2 stabilizers	/	^231^
	Compound 37		EC_50_ = 33 μmol/L	Performed extended contacts with residue at the 14-3-3/PMA2 interface, thereby stabilizing the complex with higher affinity than pyrrolidone1	/	^110^
	AMP		/	Toward the complex of 14-3-3 and the carbohydrate-response element-binding protein.	Hepatocyte	^232^
	Mizoribine		/	Enhances the interaction of glucocorticoid receptor with 14-3-3η in vitro	COS-7 cells	^233^
14-3-3 PPI inhibitors	BV02		/	Inhibit 14-3-3/c-Abl interaction and promote c-Abl nuclear translocation at low micromolar concentration	Ba/F3 cells	^234^
	FOBISIN101		IC_50_ = 9.3/16.4 μmol/L	Displaces 14-3-3–Raf-1 interactions	Human lung cancer cells	^235^
	Compound 2		/	Bound to a lysine nearby the phospho-accepting pocket of 14-3-3	Yeast cells	^236^
	2-5		IC_50_ = 2.6 μmol/L	Cross biological membranes and to disrupt 14-3-3 PPI with Raf1 and p53 without exerting cytotoxicity	mammalian cells	^237^
	Compound 15		IC_50_ = 2.6 μmol/L	Drives apoptosis in a dose-sensitive manner by activating FOXO3A-derived proapoptotic gene transcription.	DG75 leukemia cells	^238^
	Compound 8		IC_50_ = 5 μmol/L	Compound 8 inhibits viability of DG75 leukemia cells and induces apoptosis in the same concentration range	DG75 leukemia cells	^235^
	19a		IC_50_ = 5 μmol/L	Active C19a can interact with 14-3-3 proteins, such as 14-3-3θ and block their interactions with FOXO3A	Human DG-75 lymphoma cells	^239^
	BV01		/	Inhibitor of the c-Abl/14-3-3 interaction, promoted c-Abl nuclear translocation	Ba/F3 cells	^240^
	BV101		/	Inhibitor of the c-Abl/14-3-3 interaction	Ba/F3 cells	^240^
	Compound 9		/	Promote c-Abl nuclear translocation as well as to sensitize multidrug-resistant cancer stem cells	Cancer stem cells	^241^
	Xanthine		/	An endogenous modulator of 14-3-3ζ	Mouse CAD cells	^242^
	PRLX24905		IC_50_ = 50 μmol/L	Decreases the inter-action of a phosphorylated heat shock protein 20 peptide with 14-3-3	ASM cells	^243^
	UTKO1		IC_50_ = 1.98 μmol/L	Displaces 14-3-3–Raf-1 interactions	Human epidermoid carcinoma cells	^244^
	Compound B2		IC_50_ = 15 μmol/L	Phosphonate-type Inhibitors of 14-3-3. There are three unfavorable interactions with residues (Asp126, Glu133, and Glu182 of 14-3-3σ) for inhibitor binding to protein	/	^20^
	Blapsin A		/	Inhibit 14-3-3 binding to the fluorescence-labeled TMR-Raf pSer259 peptide	Mammalian cells	^245^
	Constrained peptide 18		/	An inhibitor of 14-3-3ζ	/	^109^
	Molecular Tweezers		IC_50_ = 480/520 μmol/L	The tweezers inhibit binding between the 14-3-3 protein and two partner proteins—a phosphorylated (C-Raf) protein and an unphosphorylated one (ExoS)—in a concentration-dependent manner	/	^246^
	Lacosamide		/	Directly modulate interaction with a 14-3-3 partner protein	Mouse CAD cells	^242^
14-3-3 PPI inhibitors/ Peptides	R18	/	/	Displaces 14-3-3-BAD interactions	Mouse embryonic fibroblast	^52^
	ExoS Macrocyclic Peptide	/	/	Inhibit the interaction between 14-3-3ζ and their binding partners	/	^20^
	Tau Epitope	/	/	14-3-3 proteins have been found to directly bind to Tau in solution via the phosphorylated residues Ser214 and Ser324	/	^40^
	Difopein	/	/	Blocking the ability of 14-3-3 to bind to target proteins and inhibits 14-3-3/Ligand interactions	COS-7 cells	^247^
	TVS167	/	/	Displaces 14-3-3ε–VDAC1 interactions	Mouse MA-10 Leydig cells	^248^

## 14-3-3 Proteins in bone and joint biology

**Fig. 4:**
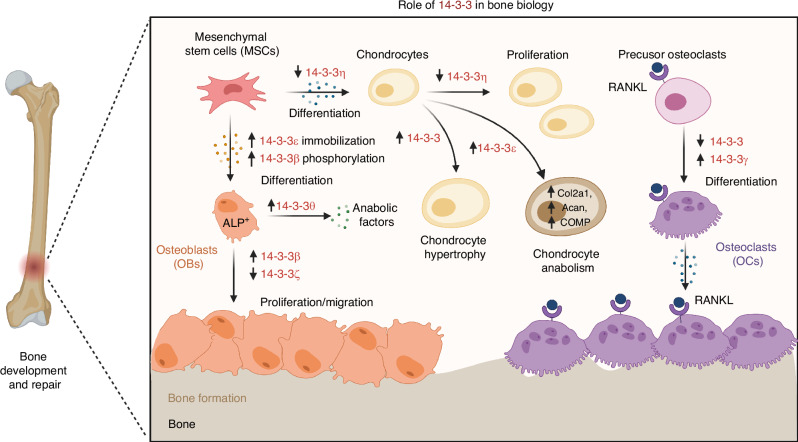
Role of 14-3-3 proteins in bone biology. The specific pathways and mechanisms by which different isoforms of 14-3-3 proteins modulate the differentiation and activity of various bone-related cells, including mesenchymal stem cells (MSCs), osteoblasts, chondrocytes, and osteoclasts, are elucidated during bone development and repair. Highlighting their involvement in key cellular processes, such as MSC differentiation, chondrocyte proliferation and hypertrophy, and osteoclast differentiation, the pathways illustrate how isoforms like 14-3-3η, 14-3-3ε, and 14-3-3γ exert their influence through mechanisms like immobilization, phosphorylation, and regulation of anabolic factors or interaction with RANKL. The interconnected nature of these cellular events ultimately contributes to bone formation and homeostasis. Figure created with BioRender.com

14-3-3 proteins regulate bone biology by interacting with various bone matrix proteins and influencing their function. Additionally, 14-3-3 proteins have been shown to be involved in the regulation of bone morphogenetic proteins, which are key regulators of bone formation and repair.^113^ Further, the importance of 14-3-3 proteins in bone biology is reviewed by emphasizing their role in key musculoskeletal cells, including osteoblasts, osteoclasts, and chondrocytes (Fig. 4).^24,114,115^

## 14-3-3 proteins in osteoblasts and osteocytes

The 14-3-3 proteins, recognized as conserved regulatory molecules, are integral to the differentiation and function of osteoblasts, the cells responsible for bone formation. Different isoforms of these proteins have been found to play specific roles in osteoblast maturation and activity. Notably, the epsilon isoform (14-3-3ε) has been shown to significantly enhance the osteogenic differentiation of human adipose-derived mesenchymal stem cells when incorporated into electrospun scaffolds.^22,116^ This is evidenced by an increase in alkaline phosphatase activity, indicating the potential of 14-3-3ε in early bone tissue regeneration and its possible application in designing scaffolds for bone repair in clinical settings.

Additionally, the theta isoform (14-3-3θ) is involved in the regulation of mechanosensitive connexin 43 hemichannels in osteocytes, which are essential for bone remodeling and integrity, particularly in response to mechanical stress.^114^ In the realm of bone pathologies, the downregulation of 14-3-3β in osteosarcoma (OS) cells correlates with decreased cell proliferation and migration, suggesting that 14-3-3β is crucial in balancing osteoblast proliferation and differentiation, and its dysregulation may lead to pathological bone formation.^27^ This makes 14-3-3β a potential therapeutic target for osteoblast-driven bone diseases.

Furthermore, the Ror2 receptor, essential for bone formation, has been found to homodimerize and induce phosphorylation of 14-3-3β, thus promoting osteoblast differentiation and bone formation.^117^ Procoxacin has been discovered through a reporter gene system to inhibit the TGF-β/C-Raf/MAPK pathway by binding to the 14-3-3ζ/C-Raf complex, thereby suppressing osteoblastic and osteoclastic activities and reducing bone metastasis in prostate cancer.^118^ These insights into the roles of 14-3-3 proteins in osteoblast biology suggest that modulating their expression or function could be key to advancing bone regenerative strategies and developing treatments for bone-related diseases.

## 14-3-3 proteins in osteoclasts

The regulation of osteoclast differentiation and function is a complex process involving numerous proteins and signaling pathways, with 14-3-3 proteins emerging as significant modulators in this context. Research has elucidated the role of 14-3-3 proteins in the regulation of osteoclast activity, particularly through their interaction with key transcription factors such as the microphthalmia-associated transcription factor (MITF). MITF is essential for osteoclast differentiation, and its activity is modulated by its cytoplasmic-nuclear shuttling, which is dependent on phosphorylation status and interactions with 14-3-3 proteins.^115^ Overexpression of 14-3-3 has been shown to retain MITF in the cytoplasm, thereby reducing the expression of osteoclast-specific genes and inhibiting differentiation.^115^ This mechanism elucidates the importance of 14-3-3 in maintaining the balance of osteoclast activity, which is crucial for bone remodeling and homeostasis.

Further investigation into the molecular interactions between 14-3-3 proteins and transcription factors has revealed that the Cdc25C-associated kinase (C-TAK1) interacts with MITF and promotes the formation of the MITF/14-3-3 complex, leading to increased cytoplasmic localization of MITF.^115^ This interaction is disrupted by RANKL/CSF-1 signaling, which is known to induce osteoclast differentiation. The dynamic regulation of MITF by 14-3-3 and C-TAK1 provides a responsive mechanism for osteoclast precursors to adapt to the bone microenvironment and differentiate accordingly. Additionally, the related transcription factor Tfe3, which does not interact with C-TAK1 or shuttle between the cytoplasm and nucleus, highlights the specificity of these regulatory mechanisms in osteoclastogenesis.^119^

The role of 14-3-3 proteins extend beyond the regulation of transcription factors to influence the broader signaling pathways involved in osteoclast regulation. Selenoprotein W (SELENOW), a protein downregulated by RANKL/RANK/TRAF6/p38 signaling, has been identified as a crucial factor in osteoclastogenesis, with its expression levels modulating osteoclast activity and bone mass (SELENOW ensures physiological bone remodeling by preventing hyperactivity of osteoclasts).^23^ SELENOW’s interaction with 14-3-3γ facilitates the nuclear translocation of key transcription factors such as NF-κB and NFATc1, thereby enhancing osteoclast differentiation. This interaction highlights the importance of 14-3-3 proteins in the regulation of osteoclasts, as their modulation could potentially serve as a therapeutic target for bone diseases characterized by excessive osteoclast activity, such as osteoporosis.

## 14-3-3 proteins in chondrocytes

The role of 14-3-3 proteins in chondrogenic differentiation has been a subject of increasing interest in the field of developmental biology. Recent studies have elucidated that 14-3-3η is notably overexpressed in the synovial fluid of patients with joint inflammation.^120^ 14-3-3 overexpression correlates with a reduction in chondrogenic differentiation, as demonstrated in ATDC5 cells. The presence of inflammatory cytokines such as TNF-α further exacerbates the overexpression of 14-3-3η, leading to the inhibition of chondrogenesis.^24^ Conversely, silencing of 14-3-3η has been shown to promote chondrogenic differentiation, suggesting a potential therapeutic target for enhancing chondrocyte maturation in pathological conditions. In addition to its inhibitory role, 14-3-3η has been implicated in the cell cycle regulation of chondrocytes. Overexpression of 14-3-3η was found to prevent G1 phase arrest, a critical checkpoint for cell proliferation and differentiation.^24^ This finding indicates that 14-3-3η not only affects the differentiation of chondrocytes but also their proliferative capacity, which is essential for proper growth plate development and function. The precise mechanisms by which 14-3-3η modulates these processes remain to be fully elucidated, but the evidence points to a multifaceted role in chondrocyte cell cycle and differentiation pathways.

Furthermore, the interaction between histone deacetylase 4 (HDAC4) and 14-3-3 proteins has been identified as a critical regulatory axis in cell differentiation.^121,122^ HDAC4, which is known to reside in the nucleus of proliferating chondrocytes, relocates to the cytoplasm during the transition to the prehypertrophic stage, a process regulated by Ca^2+^/calmodulin-dependent kinase IV (CaMKIV) signaling.^123^ CaMKIV not only facilitates the cytoplasmic relocation of HDAC4 but also its phosphorylation, enabling its association with 14-3-3 proteins. This interaction is crucial for the activation of key transcription factors such as Runx2 and the expression of differentiation markers like type X collagen (Col X) and Indian hedgehog (Ihh), driving the maturation of chondrocytes.^123^ The intricate relationship between CaMKIV, HDAC4, and 14-3-3 proteins underscore the complexity of the regulatory networks governing chondrocyte differentiation and highlights potential targets for therapeutic intervention in growth-related disorders.

## 14-3-3 proteins in bone remodeling

The intricate network of bone remodeling is orchestrated by a multitude of factors, among which the 14-3-3 proteins have been identified as key regulators. These proteins, known for their ability to bind serine/threonine-phosphorylated proteins, are now recognized for their specific roles in bone physiology.^124^ For instance, 14-3-3θ has been shown to facilitate the plasma membrane delivery and function of mechanosensitive connexin 43 hemichannels in osteocytes, a process that is essential for bone formation and remodeling in response to mechanical stress.^114^ This scaffolding role of 14-3-3θ is critical for the interaction between connexin 43 and integrin α5, which is necessary for the opening of hemichannels upon mechanical loading, highlighting the importance of 14-3-3 proteins in the mechanotransduction pathway.^114^ In addition to their role in mechanotransduction, 14-3-3 proteins are involved in the regulation of osteoclast differentiation and osteoblastogenesis. SELENOW, which interacts with 14-3-3γ, is downregulated during osteoclast differentiation and is essential for preventing the hyperactivity of osteoclasts, thus ensuring physiological bone remodeling.^23^ Conversely, the Ror2 receptor, which is crucial for bone formation, has been found to homodimerize and phosphorylate 14-3-3β, suggesting that 14-3-3β may act as a negative regulator of osteogenesis.^117^ The phosphorylation of 14-3-3β by Ror2 could potentially release the inhibition of osteoblast differentiation, promoting bone formation.

Furthermore, the interaction of the parathyroid hormone receptor (PTHR) with 14-3-3 proteins suggests a regulatory role in PTHR signaling, which is vital for calcium homeostasis and bone remodeling.^25^ 14-3-3 proteins facilitate the interaction between HDAC4 and cytoplasmic compartments, influencing HDAC4’s nuclear translocation, which is crucial for repressing the transcriptional activity of MEF2, a key regulator of chondrocyte hypertrophy.^125^ Parathyroid hormone-related protein (PTHrP)/forskolin-induced dephosphorylation of HDAC4 at phospho-S246 reduces its interaction with 14-3-3 proteins, promoting its nuclear translocation and subsequent repression of MEF2 transcriptional activity, ultimately contributing to the regulation of chondrocyte hypertrophy during bone remodeling.^118,119^ These findings collectively underscore the multifaceted roles of 14-3-3 proteins in bone remodeling, from subcellular signaling to the regulation of gene expression and protein trafficking, offering new insights into potential therapeutic targets for bone-related diseases.

## 14-3-3 proteins in fibroblast-like synoviocytes

Fibroblast-like synoviocytes (FLS) play a vital role in the pathogenesis of RA and OA by producing proinflammatory factors, inflammatory mediators, and metalloproteinases that lead to joint inflammation and cartilage destruction.^126^ Within the 14-3-3 protein family, the isoforms 14-3-3η and 14-3-3ε play significant roles in these conditions. In RA, 14-3-3η is particularly important as it promotes the formation of invadosomes in FLSs via the FOXO3-Snail axis.^127^ This process enhances the degradation of extracellular matrix components, leading to joint damage. Mechanistic studies have shown that knocking down 14-3-3η results in a significant reduction in invadosome formation. Conversely, introducing recombinant 14-3-3η protein to FLSs from healthy individuals induces invadosome formation, highlighting the critical role of 14-3-3η in cartilage degradation and joint damage in RA. In OA, 14-3-3ε functions as an alarmin, an endogenous molecule that perpetuates inflammation by activating the innate immune system.^30^ It interacts with Toll-like receptors 2 (TLR2) and 4 (TLR4), leading to macrophage polarization and an inflammatory phenotype in synoviocytes, macrophages, and chondrocytes. Experimental studies have demonstrated that 14-3-3ε stimulates the expression and release of inflammatory mediators such as IL-6 and monocyte chemoattractant protein-1 in these cells.^30^ Notably, the inflammatory effects of 14-3-3ε are significantly reduced when TLR2 and TLR4 are inhibited or knocked out, highlighting the essential role of 14-3-3ε in synovial inflammation. Therefore, targeting 14-3-3 proteins, particularly 14-3-3η and 14-3-3ε, presents a promising therapeutic strategy for reducing joint inflammation and destruction in joint-related diseases.

## Interaction with bone-related pathways

The 14-3-3 family of proteins has emerged as a pivotal regulator of bone-related pathways, integrating multiple signaling cascades that are essential for bone development and disease. These proteins act as scaffolds or adaptors, forming complexes that modulate signal transduction and cellular responses. For instance, the 14-3-3zeta isoform is phosphorylated on Tyr(179) in response to cytokines like granulocyte-macrophage colony-stimulating factor, which enables it to bridge phosphoserine and phosphotyrosine signaling.^128^ In hematopoiesis, 14-3-3 proteins modulate the LNK/JAK2 pathway in mouse HSPCs, disrupting the LNK-JAK2 interaction to alleviate LNK’s inhibition of JAK2 signaling, thus promoting cell proliferation and HSPC reconstitution.^129^ Regarding bone health and disease, 14-3-3 proteins influence several pathways. They modulate Wnt/β-catenin signaling, evidenced by the inhibitory effect of 14-3-3β downregulation on OS cell proliferation and migration.^27^ The miR-204/14-3-3ζ axis regulates OS cell proliferation via the STAT3 pathway, suggesting therapeutic potential for targeting 14-3-3-mediated pathways in OS.^130^

14-3-3 proteins also facilitate BMP and Wnt pathway crosstalk by aiding β-catenin activation by Akt in intestinal stem cells.^113^ Additionally, they regulate LFA-1-dependent inflammatory cell recruitment by modulating the LFA-1 and Cbl-b interaction, affecting immune cell adhesion and migration.^131^ Furthermore, 14-3-3 proteins interact with MITF in osteoclast precursors, affecting osteoclast differentiation by promoting MITF’s cytosolic localization.^115^ Ror2 receptor-mediated phosphorylation of 14-3-3β promotes osteogenesis, indicating a negative regulatory role of 14-3-3β in this process.^117^ In chondrocyte hypertrophy, essential for endochondral bone formation, 14-3-3 proteins participate in the PTHrP/cAMP/PKA signaling pathway, leading to decreased phosphorylation of HDAC4, permitting its nuclear translocation and inhibition of hypertrophy.^125^ These findings collectively underscore 14-3-3 proteins are pivotal regulators of multiple signaling pathways, including PI3K/Akt, JAK2, Wnt/β-catenin, STAT3, and PTHrP/cAMP/PKA, integral to cell survival, hematopoiesis, bone health, immune response. Therefore, 14-3-3 proteins are integral to the complex network of interactions that govern bone biology, and further research is needed to fully understand their specific roles and mechanisms of action.

## 14-3-3 proteins in bone and joint-related diseases

### Osteoarthritis

OA is a prevalent joint disorder and a chronic degenerative disease associated with aging, characterized by cartilage degeneration, bone remodeling, osteophyte formation, and joint inflammation.^132–135^ Treatment options for OA are limited, primarily involving analgesics and non-steroidal anti-inflammatory drugs which only offer symptomatic relief.^136^ In case of advanced disease, patients may resort to joint replacement surgery, which, despite providing relief, carries the risk of complications and the prosthetics have finite lifespans.^137^ The increasing incidence of OA, particularly among the elderly, imposes significant health and economic burdens, underscoring the urgent need for novel treatment strategies. Chondrocytes, the sole cell type in cartilage, produce the extracellular matrix comprising type II collagen, hyaluronic acid, and proteoglycan matrices, which are crucial for joint homeostasis.^138,139^ The limited regenerative capacity of chondrocytes and a pro-inflammatory environment that impairs chondrocyte function and promotes apoptosis are primary contributors to this degeneration. Targeting chondrocyte function could therefore represent a therapeutic strategy for OA.^140^

14-3-3 proteins, known to bind various phosphorylated client proteins, regulate cell cycle and apoptosis.^141^ Early research indicated that Clematis mandshurica extract could inhibit staurosporine-induced apoptosis in rat chondrocytes by upregulating 14-3-3 for its anti-apoptotic function. Specifically, 14-3-3 knockdown negated the extract’s protective effects against apoptosis, highlighting the protein’s role in chondrocyte survival.^141,142^ Further investigations have demonstrated that 14-3-3 binds to phosphorylated Ser112 on Bad, preventing its interaction with Bcl-xL and promoting cell survival.^136^ These findings suggest a potential anti-apoptotic role for 14-3-3 in chondrocytes but fall short of elucidating the underlying mechanisms and lack in vivo data to confirm 14-3-3’s role in OA.

Our recent study reveals that 14-3-3ε acts as a crucial component of the TNFR2 receptor complex in chondrocytes, modulating progranulin-mediated protection against OA progression, thus highlighting a novel pathway with therapeutic implications for the disease.^29^ RNA sequencing of cartilage from OA and non-OA patients showed decreased expression of 14-3-3ε, in OA-affected cartilage. This finding was corroborated in a medial meniscus destabilization model, with reduced mRNA and protein levels of 14-3-3ε observed in cartilage from OA mice. To clarify 14-3-3ε‘s role in OA, we generated 14-3-3ε-deficient mice, which exhibited OA-like phenotypes, such as proteoglycan loss and decreased articular cartilage thickness. Similarly, mice with cartilage-specific deletion of 14-3-3ε developed more severe OA symptoms post-DMM surgery.^29^ In vitro studies further showed that 14-3-3ε knockout in C28/I2 cells via CRISPR-Cas9 reduced the anabolic response to PGRN and exacerbated the catabolic response to TNF-α, changes that were reversible with 14-3-3ε re-expression.^29^ The signaling through TNFR2/14-3-3ε, activated by PGRN, involves the ERK-dependent activation of Elk-1 and suppression of NF-κB, delineating a protective pathway against OA. Additionally, 14-3-3ε knockout mice displayed an altered M1/M2 macrophage ratio, favoring an inflammatory phenotype.^26^

The role of 14-3-3 in OA is multifaceted. While some studies, including our own, suggest that isoforms of 14-3-3 slow OA progression, others report a role in hastening it. Among many possible factors, the use of different approaches may contribute to the inconsistency reported. For instance, some labs employed recombinant 14-3-3 protein, which is expected to function as an extracellular factor, whereas others utilized gene deletion models that primarily focus on the role of intracellular 14-3-3 proteins. Joint homeostasis involves various cells such as chondrocytes, synoviocytes, immune cells, osteoblasts, and osteoclasts, with joint destruction being the cumulative effect of multiple cellular activities. Investigating the roles of different cells and their interconnections in joint destruction could identify therapeutic targets.^143^ One study demonstrated the catabolic phenotype of chondrocytes treated with conditioned medium from compressed osteoblasts/osteocytes, highlighting the role of 14-3-3ε as a mediator released under mechanical stress.^143^ Specifically, osteoblasts/osteocytes under mechanical stress induced elevated MMP3, MMP13 expression and decreased type II collagen, aggrecan expression in chondrocytes by releasing 14-3-3ε. Notably, 14-3-3ε lacks a receptor in chondrocytes, but it has been shown to bind to CD13 on cartilage surface, and inhibiting CD13 can significantly reduce 14-3-3ε-induced MMP3 mRNA expression and protein release.^144^ In vitro models using human macrophages and synovial explants from OA patients have shown that recombinant 14-3-3ε skews these cells towards a pro-inflammatory phenotype.^30^ CD13 also serves as a receptor for 14-3-3σ in FLS, involved in fibroblast epithelial-mesenchymal transformation and MMP1 release.^145^

**Fig. 5:**
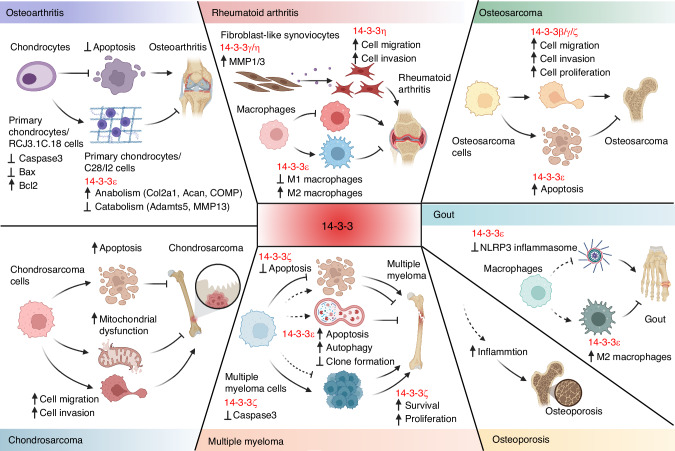
14-3-3 proteins in bone and joint-related diseases. In different cell and animal models, abnormal expression levels of 14-3-3 protein can be used as biomarkers for diseases such as osteoarthritis, rheumatoid arthritis, multiple myeloma, gout, osteoporosis, and bone tumors, and also participate in regulating the disease process. Therefore, appropriate modulation of 14-3-3 and their protein-protein interactions may be a potential strategy for treating bone and joint-related diseases. Figure created with BioRender.com

Despite the growing understanding of 14-3-3ε‘s role in OA, evidence on the involvement of other 14-3-3 isoforms remain scant. The varied functions of these isoforms in different cell types and physiological contexts are not consistent, and their roles at different OA stages requires further exploration. Intriguingly, clinical data suggest a link between 14-3-3 and OA pathogenesis, with a specific microRNA in OA patient plasma accelerating OA progression and appearing closely related to 14-3-3. Additionally, 14-3-3ε is associated with a poor prognosis for cartilage repair post-osteotomy.^146,147^ Yet, there are currently no 14-3-3-targeted drugs in clinical trials for OA, highlighting the need for continued research to establish a definitive link between 14-3-3 and OA (Fig. 5).

Traditionally, OA was considered a localized joint lesion. However, biomarker studies reveal systemic changes during OA development. Researchers observed significant changes in sugar metabolites in the blood and synovial fluid of OA patients.^148^ An epidemiologic survey of 25 514 volunteers indicated that the triglyceride glucose (TyG) index directly correlates with OA risk, with each incremental unit increase in the TyG index associated with a 634% increase in risk.^149^ Similarly, elevated blood glucose exacerbates OA symptoms.^150^ *YWHAB* regulates the glucagon receptor (GCGR). Mechanistic studies show *YWHAB* forms a phosphorylation-dependent complex with GCGR and interacts with FOXO1 to inhibit hepatic gluconeogenesis.^151^ Notably, 14-3-3 is implicated in diabetes pathogenesis. Studies demonstrate that OA patients with diabetes and cardiovascular disease experience accelerated cartilage degeneration.^152^ In a diabetic cardiomyopathy model, Ask1 activation in 14-3-3η heart-specific knockout mice significantly impaired cardiac function.^153^ Whether 14-3-3 involvement in diabetic cardiomyopathy further affects OA requires experimental evidence. Additionally, obesity is a risk factor for OA. 14-3-3ζ, an adaptor protein regulating insulin signaling in lipolysis and synthesis, influences obesity. 14-3-3ζ knockout mice are lean with reduced visceral fat, while 14-3-3ζ overexpression exacerbates obesity.^154^ Mechanistic studies show 14-3-3ζ depletion promotes autophagy-dependent C/EBP-δ degradation, preventing induction of adipogenic factors PPARγ and C/EBP-α. Since obesity exacerbates cartilage wear, 14-3-3 may be involved in OA development by regulating body weight. However, direct experimental evidence for this hypothesis is lacking.

### Rheumatoid arthritis

RA is a chronic systemic autoimmune disease characterized by synovial cell proliferation, inflammatory cell infiltration, pannus formation, and bone erosion.^6,155,156^ RA’s pathogenesis is multifaceted, involving genetic, epigenetic, and environmental factors, and despite advancements in treatment over the past three decades, a cure remains elusive.^157^ Thus, identifying new therapeutic targets is crucial for the future management and prevention of RA.

In a population-based study, RA patients exhibited elevated serum levels of 14-3-3η protein at 3.26 ng/mL, a stark contrast to the 0.37 ng/mL in healthy controls.^158^ These levels correlated positively with baseline radiographic damage, suggesting 14-3-3η as a potential RA biomarker.^159^ The question then arises: what is the specific role of 14-3-3η in RA, and what is the source of 14-3-3 in RA patient serum and synovial fluid? Research has shown that 14-3-3η is highly expressed in synovial tissue macrophages,^31^ and in vitro assays have revealed that TNF-α activates macrophage secretion of 14-3-3η, likely through inducing a necroptosis-like cell death, releasing 14-3-3η extracellularly.^160^ 14-3-3 secreted by synovial macrophages may be received by synovial fibroblasts, as increased 14-3-3 expression was found in fibroblasts in the same synovial tissue. Moreover, 14-3-3 is an inducer of FLS migration and invasion. 14-3-3 secreted in serum or synovial fluid has been considered as a biomarker for RA diagnosis.^161^ Clinically, TNFα inhibitors like ADA have been observed to reduce serum 14-3-3η levels in RA patients,^160^ further suggesting that 14-3-3η may serve as a potential target molecule for RA.

The synovium plays a critical role in joint homeostasis. Synovial inflammation, hyperplasia, and the invasion of synovial cells into cartilage are pivotal in RA pathogenesis, leading to the destruction of cartilage and bone.^162–164^ Therefore, targeting synovium-associated pathologies is a strategic treatment approach in RA. Immunohistochemical analyses have revealed increased 14-3-3η expression in the synovial tissue of RA patients compared to those with OA,^127^ with FLS from RA patients also expressing higher 14-3-3η levels, correlating with the degree of 14-3-3 expression. To elucidate the relationship between 14-3-3η and FLS migration and invasion, studies employing human recombinant 14-3-3η protein and 14-3-3η knockdown treatments were conducted. The findings indicated that recombinant 14-3-3η enhanced FLS migration, whereas knockdown had the opposite effect. Mechanistic investigations showed that 14-3-3η facilitated FOXO3 nuclear export and activated the transcription factor Snail, known for regulating tumor metastasis, to promote FLS cell migration.^127^

The discovery of 14-3-3ε as a pivotal regulator in the pathogenesis of RA adds a new dimension to our understanding of this debilitating disease. Our recent findings elucidate the role of 14-3-3ε in macrophage polarization, a critical determinant in the balance of pro-inflammatory (M1) and anti-inflammatory (M2) macrophage phenotypes.^26^ The myeloid-specific deletion of 14-3-3ε was observed to exacerbate collagen-induced arthritis (CIA), suggesting a protective role of 14-3-3ε in the inflammatory milieu of RA by favoring the M2 phenotype over the M1 phenotype.^26^ A key aspect of this regulation is the interaction of 14-3-3ε with TNFR2, a receptor known for its anti-inflammatory and immunomodulatory properties.^106,165,166^ Our data indicate that 14-3-3ε is integral to the TNFR2 receptor complex and essential for mediating the receptor’s anti-inflammatory signals. This interaction appears to activate the PI3K/Akt/mTOR pathway, which in turn inhibits NF-κB activation and promotes C/EBPβ activation, leading to a shift towards anti-inflammatory macrophage polarization.^26^ Furthermore, the anti-inflammatory actions of PGRN, which serves as a ligand for TNFR2, are markedly diminished in the absence of 14-3-3ε. This reduction in PGRN’s efficacy was particularly pronounced in the context of inflammatory arthritis, where PGRN’s inflammation-suppressing effects were compromised in mice with a myeloid-specific deficiency in 14-3-3ε.^26^ These insights collectively point to the TNFR2/14-3-3ε signaling axis as a crucial modulator of macrophage plasticity and reveal a promising therapeutic target for immune modulation in RA. The therapeutic implications of these findings hold substantial promise for the development of targeted interventions aimed at restoring immune homeostasis in RA patients.

While there is no direct evidence yet of 14-3-3 involvement in RA cartilage degradation, extensive research indicates its role in promoting anabolism in OA chondrocytes or protecting against cartilage degradation. Beyond 14-3-3η, elevated levels of 14-3-3γ have also been detected in synovial fluid, correlating positively with MMP1 and MMP3 expressions, key factors in FLS invasion.^167^ However, experimental evidence linking 14-3-3γ to FLS migration and invasion is lacking. Therefore, the specific role of 14-3-3 in RA pathology warrants further investigation. Encouragingly, 14-3-3 proteins, particularly 14-3-3ε and 14-3-3η, have emerged as crucial players in the immune and inflammatory responses associated with RA. The pivotal role they play in synovial inflammation, macrophage polarization, and FLS behavior presents new opportunities for targeted therapeutic interventions aimed at modulating these pathways and restoring immune homeostasis in RA patients. These findings provoke a reevaluation of traditional RA management strategies and pave the way for more personalized and effective treatment modalities (Fig. 5).

### Bone-related tumors

The 14-3-3 protein family has emerged as a significant regulator in tumorigenesis, marking it as a promising target for tumor therapy.^168^ These proteins also appear to be pertinent in bone-related tumors, such as OS and synovial sarcoma (Fig. 5). Given its propensity to lead to bone destruction, multiple myeloma (MM), though traditionally not classified as a bone-related tumor, is also reviewed in this context.

#### Osteosarcoma

OS is the foremost malignant bone tumor, characterized by persistent bone pain and a high tendency for metastasis and recurrence, especially in the lungs, complicating treatment.^169–171^ The high metastasis rate of OS results in patients with low 5-year survival, making the search for strategies to target OS metastases the most critical challenge.^170^ The 14-3-3 protein family’s role in tumor progression has been extensively studied, with different isoforms implicated in the migration and invasion of various cancer cells.^27,172–174^ These findings suggest a potential involvement of 14-3-3 proteins in OS metastasis, warranting further investigation into their viability as therapeutic targets for OS.

Elevated expression of 14-3-3β in OS tissues and cell lines indicates a positive correlation with OS aggressiveness.^27^ Knockdown studies of 14-3-3β have shown reductions in OS cell proliferation, migration, and invasion, underscoring its significance in OS pathophysiology. Yet, the specific mechanisms by which 14-3-3β influences these processes remain to be elaborated. Other isoforms, such as 14-3-3γ and 14-3-3ζ, have also been implicated in OS progression, with studies suggesting their involvement in cell proliferation and migration via the MicroRNA-222/*YWHAG* and miR204/14-3-3ζ/STAT3 pathways, respectively.^130,175^ Interestingly, 14-3-3ε appears to enhance chemosensitivity in OS by promoting BIM expression, although this effect is mitigated by pERK1/2.^176^ These insights into the diverse roles of 14-3-3 isoforms disclose the complexity of their function in OS and the potential for targeted therapeutic strategies.

Blood glucose and obesity are also thought to be associated with the prognosis of OS. Studies have shown that both primary and metastatic OS tissues show positivity for Glucose transporter protein-1 (Glut-1), a key protein in glucose metabolism, suggesting increased glucose uptake in OS.^177^ 14-3-3 regulates glucose uptake by interacting with AKT-AS160 phosphorylation, affecting Glut-1.^178^ Regarding obesity and OS, the fat mass and obesity-associated protein induces OS migration and invasion.^179^ However, as with OA, direct evidence for 14-3-3 involvement in OS development through the regulation of obesity or blood glucose is lacking.

#### Chondrosarcoma

Chondrosarcoma, ranking second to OS among primary malignant bone tumors, exhibits a wide prognostic spectrum based on its grade.^180^ Chondrosarcoma (CS) arises within the bone and is usually defined by the development of a chondroid matrix, occurring at an approximate annual rate of 1 per 200 000 individuals.^181,182^ Treatment options are limited, with surgical resection being the primary choice for low-grade tumors, while high-grade tumors pose a greater risk of metastasis and recurrence.^183–185^ The search for novel therapeutic targets is therefore of paramount importance. The potential role of 14-3-3 proteins in CS is evidenced by the studies demonstrating their involvement in tumor malignancy. For instance, EGCG’s induction of apoptosis in CS cells was linked to the modulation of the 14-3-3/ASK1 complex.^186^ However, further in vivo and in vitro studies targeting 14-3-3 are required to validate these findings.

The 14-3-3 family’s role in the regulation of EMT is well-documented, implicating it in CS metastasis.^187–190^ For example, 14-3-3ε has been shown to enhance TGF-βRI-mediated TGF-β signaling,^191^ while 14-3-3σ is activated by the TGF-β1/SMAD3 pathway.^192^ The relationship between 14-3-3 and the TGF-β pathway, particularly in the context of an identity switch in tumor stages, is an area of great interest.^193^ Additionally, 14-3-3 proteins’ interaction with EMT-associated transcription factors, such as Slug, suggests a nexus between 14-3-3 regulation and tumor progression.^194,195^ Despite limited direct evidence, the established role of 14-3-3 in other tumor types and its regulation of key metastatic signaling pathways provide a rationale for further exploration of its therapeutic potential in CS.

#### Multiple myeloma

MM is a hematological malignancy characterized by the uncontrolled proliferation of clonal plasma cells, leading to significant bone damage.^196,197^ The increasing incidence of MM, especially among the elderly, underscores the necessity for innovative therapeutic targets and strategies.^198,199^ The role of 14-3-3 proteins in MM is suggested by their abnormal expression levels and association with telomere maintenance, a key factor in MM cell viability.^200^ While high 14-3-3 expression correlates with stable telomere lengths, the precise mechanisms by which 14-3-3 proteins contribute to telomere stabilization in MM require further clarification.

Proteasome inhibitors are a cornerstone of MM treatment, yet resistance remains a challenge.^201,202^ The expression of 14-3-3δ not only serves as a prognostic marker but also influences proteasome assembly, impacting MM cell viability.^10,203^ The sensitivity of MM cells to proteasome inhibitors appears to be modulated by 14-3-3ε, with knockdown studies revealing altered responses to treatment.^204^ These findings uncover the importance of elucidating the roles and mechanisms of 14-3-3 isoforms in MM to inform the development of targeted therapeutics.

## Gout

Gout arises from a complex interplay of genetic predisposition and lifestyle factors that lead to hyperuricemia and subsequent deposition of monosodium urate (MSU) crystals in joints and tissues, triggering acute and intense inflammatory episodes.^205^ As gout is prevalent in the older population and associated with cardiovascular and chronic kidney diseases, the aging population is experiencing an increasing burden of this condition.^205,206^ Recent studies have revealed elevated serum levels of 14-3-3η protein in patients with gout compared to healthy individuals, suggesting a potential biomarker role for this protein in the disease.^207^ The diagnostic value of 14-3-3η is supported by its significant area under the receiver operating characteristic curve, indicating a notable distinction between gout patients and healthy controls.^207^ However, the literature on this topic is scarce, emphasizing the need for additional research.

The pathogenesis of gout is closely linked to the activation of the NLRP3 inflammasome, a key component in the inflammatory response to MSU crystals.^208,209^ This activation leads to the processing of pro-inflammatory cytokines IL-1β and IL-18, which are instrumental in the clinical manifestations of gout.^208,210–212^ Interestingly, 14-3-3 proteins, particularly 14-3-3ε, have been implicated in the regulation of the NLRP3 inflammasome and in macrophage polarization towards an anti-inflammatory M2 phenotype.^15,26^ Although direct evidence implicating 14-3-3 proteins in gout is not extensive, the proteins’ established role in inflammation, and the inflammatory nature of gout, imply a potentially important role for 14-3-3 in the disease. Further research is warranted to elucidate this role and to determine whether 14-3-3 proteins could serve as effective biomarkers or therapeutic targets in gout (Fig. 5).

## Osteoporosis

Osteoporosis represents a systemic skeletal disorder characterized by reduced bone mass and microarchitectural deterioration of bone tissue, leading to increased fragility and fracture risk.^213,214^ Osteoporosis is most prevalent in the elderly, with a high mortality rate from hip fractures caused by osteoporosis.^215–217^ With the aging of the population, the social health costs caused by osteoporosis have risen dramatically.^218,219^ Early diagnosis and effective treatment strategies are therefore urgently needed.

Previous research has indicated that the expression of 14-3-3 proteins is significantly upregulated in the serum of patients with RA and may serve as a diagnostic marker.^158^ This prompts the question of whether 14-3-3 proteins could also be indicative of osteoporosis. In the context of RA-related osteoporosis, 14-3-3η has been identified as an independent risk factor, with higher serum levels noted.^158^ Yet, contrasting findings have been observed in a serum follicle-stimulating hormone-induced postmenopausal osteoporosis rat model, where 14-3-3η expression was downregulated and inversely correlated with bone trabecular area.^220^ This discrepancy may stem from the fact that RA-related osteoporosis is primarily inflammation-driven, which could account for the elevated levels of 14-3-3η.^28,221^

Direct evidence of the role of 14-3-3 proteins in osteoporosis remains scarce. However, the involvement of NF-κB and ERK signaling pathways in osteoporosis pathogenesis^222–224^ and their known association with 14-3-3 proteins suggest a potential regulatory role.^225,226^ The similarity in mechanisms through which 14-3-3 proteins modulate pathological processes in other diseases to those in osteoporosis, together with findings in animal models, supports the hypothesis that 14-3-3 proteins could serve as both prophylactic biomarkers and therapeutic targets for osteoporosis. Nevertheless, delineating the specific role of 14-3-3 in osteoporosis pathophysiology remains a formidable challenge (Fig. 5).

## Future directions

The current clinical evidence positions 14-3-3 proteins as pivotal players in the pathology of musculoskeletal disorders, with a particular focus on RA and OA (Table 2). Elevated levels of isoforms such as 14-3-3η in RA patients’ synovial fluid and serum have been linked to disease activity and joint damage, indicating their potential as biomarkers. This isoform’s role in promoting inflammatory cytokines and metalloproteinases suggests a direct involvement in cartilage degradation, making it a compelling target for therapeutic intervention. Similarly, in OA, the 14-3-3ε isoform has been associated with chondrocyte homeostasis disruption and matrix degradation. These findings underscore the potential of 14-3-3 proteins not only as biomarkers for disease stratification and prognosis but also as targets for personalized medicine, where treatments could be tailored to the patient’s specific 14-3-3 profile, potentially improving clinical outcomes in bone and joint diseases.

**Table 2:** The role of the 14-3-3 protein in bone and joint-related diseases

Disease	Subunit	Substances	Experimental cells	Effect on disease (in vitro)	Clinical specimens or experiment model	Effect on disease (in vivo)	Ref
OA	14-3-3	14-3-3 KD	RCJ3.1C.18, Rat chondrocytes	Bcl2↓, Bclxl↓, Caspase-3↑, cell viability↓, Bax↑			^136^
	14-3-3ε	14-3-3ε KO	C28I2, human or mouse primary chondrocytes	Col2a1↓, Acan↓, COMP↓, Adamts5↑, MMP13↑, Cox2↑, Nos2↑	Age-related model, DMM model	OARSI scores↑, proteoglycan content↓, cartilage thickness↓, subchondral bone and osteophyte formation↑, aggrecan neoepitope↑, COMP fragments↑, ColX↑, MMP13↑, Adamts5↑, Cox-2↑	^29^
	14-3-3ε	Recombinant 14-3-3ε	Mouse chondrocytes	MMP3 and MMP13 proteins↑, Type II collagen and aggrecan mRNA↓			^147^
	14-3-3ε	Recombinant 14-3-3ε	Synovial explants, Human FLS	IL6↑, MCP1↑			^30^
			Mouse articular chondrocytes	MMP3↑, IL6↑			
			BMDMs, THP-1 cell line	IL6↑, MCP1↑, TNF-α↑			
RA	14-3-3ε	14-3-3ε KO	BMDMs, Raw264.7	M1 polarization↑(IL6↑, NOS2↑), M2 polarization↓(Arg1↓, Mgl1↓)	CIA model	Joint swelling↑, inflammation↑, clinical score↑, bone and cartilage destruction↑, IL-6↑, TNF-α↑, osteoclasts↑, Treg cells↓, IL-10↓	^26^
	14-3-3η				Patient’s serum and SF	Baseline radiographic damage↑	^159^
	14-3-3η	TNF-α	Primary macrophages	Necroptosis↑, p-MLKL↑			^31^
	14-3-3η	Recombinant 14-3-3η	Human FLS	Invadosome formation↑, cortactin↑, actin↑, Snail↑			^127^
OS	14‑3‑3β	14-3-3β KD	U2OS, Saos-2 and MG63	Cell viability↓, cell cycle progression↓, cell invasion↓			^27^
	14-3-3γ	14-3-3γ-OE	Saos2, 143B, HOS, MG63, U2OS	Cell viability↑, cell invasion↑			^175^
	14-3-3ζ	14-3-3ζ KD	143B, Saos2, MG63 and U2OS	Cell viability↓, colony formation↓			^130^
	14-3-3ε	14-3-3ε KD	143B	Apoptosis↓			^176^
CS	14-3-3		JJ012	Apoptosis↑			^186^
MM	14-3-3		JIM-1, KMS-11, KMS12, KMS-26, KMS-28, MM1R, RPMI8226 and U266	Stable short lengths of telomeres↑			^200^
	14-3-3ζ	14-3-3ζ KD	MM stable cell lines	Proliferation↓, proteolytic function↓, cell death↑			^203^
	14-3-3ε	14-3-3ε KD	Human primary MM cells	Cell viability↓			^249^
		14-3-3ε-OE			Xenograft model	Tumor burden↓	
Gout	14-3-3η				Patients’ serum	14-3-3η↑	^207^
Osteoporosis	14-3-3η				Patients’ serum	14-3-3η↑	^158^
	14-3-3η				FSH-induced postmenopausal osteoporosis rat model	14-3-3η↓, bone trabecular area↓	^220^

↑ increase, ↓ decrease, *OA* osteoarthritis, *DMM* surgical destabilization of the medial meniscus, *RA* rheumatoid Arthritis, *BMDMs* bone marrow-derived macrophages *CIA* collagen-induced arthritis, *OS* osteosarcoma, *CSn* chondrosarcoma, *MM* multiple myeloma, *FLS* fibroblast-like synoviocytes, *FSH* follicle-stimulating hormone, *SF* synovial fluid, *KD* knockdown, *KO* knockout, *OE* over-expression

Despite the promising therapeutic potential of 14-3-3 proteins, several challenges and obstacles remain. The highly conserved nature of their phospho-binding pockets complicates the development of isoform-specific inhibitors, a necessary step given the distinct roles of each isoform in various cellular processes. While some compounds have shown specificity for the *ζ* isoform, the underlying mechanisms of this selectivity are not fully understood, and the potential for functional compensation by other isoforms raises concerns about the efficacy of targeting a single isoform in disease treatments. Furthermore, the accuracy of 14-3-3 detection as a clinical diagnostic indicator is not yet optimal. The timing and means of detection require further exploration and verification to ensure that 14-3-3 proteins can reliably serve as biomarkers for monitoring disease severity and treatment response. Addressing these challenges will necessitate a multifaceted approach, combining advanced molecular design, sensitive diagnostic tools, and comprehensive longitudinal studies.

The future prospects for 14-3-3 protein research and application are bright and varied. As more binding partners are identified, the involvement of 14-3-3 proteins in vital cellular processes is expected to become clearer, potentially revealing new therapeutic targets. The pursuit of small molecule inhibitors and/or stabilizers that can modulate 14-3-3 interactions globally or with isoform specificity will likely advance our understanding of their function and open up new avenues for drug development. In regenerative medicine, the modulation of 14-3-3 proteins could enhance osteoblast/chondrocyte differentiation and bone/cartilage formation or inhibit osteoclast activity, offering innovative solutions for bone regeneration and cartilage repair. The integration of pharmacological, animal, and clinical studies will be crucial in translating these insights into effective treatments for musculoskeletal and other diseases, ultimately contributing to the advancement of personalized medicine and improving patient care.

Additionally, artificial intelligence (AI) has significant potential in 14-3-3 research and drug screening. By analyzing large-scale proteomic and genomic datasets, AI can identify new 14-3-3 protein interactions and binding partners, predicting their functional consequences and key pathways affected by 14-3-3 proteins. In drug discovery, AI-driven platforms can accelerate the screening of potential inhibitors or stabilizers by predicting their binding affinity and specificity for different 14-3-3 isoforms, reducing the time and costs of traditional drug development. AI can also enhance the precision of diagnostic tools by improving the sensitivity and specificity of assays detecting 14-3-3 proteins in biological samples. Advanced image analysis algorithms can quantify 14-3-3 protein expression in tissue samples, allowing more accurate assessment of disease severity and treatment response. This synergy between AI and molecular biology can significantly enhance our understanding and treatment of bone and joint-related diseases, paving the way for innovative and personalized healthcare solutions.

## Conclusion

14-3-3 proteins have transcended their original classification as mere regulatory components, now recognized for their versatile roles in signal transduction and intricate protein interactions. These proteins are essential in modulating cellular processes that underpin bone and joint health, identifying them as promising candidates for biomarkers and therapeutic agents across a spectrum of pathologies. Notably, variations in the levels of the 14-3-3 isoform have been correlated with the progression of joint diseases, emphasizing its utility for early detection and as a predictor of disease trajectory. The potential applications of 14-3-3 proteins in treating bone and joint diseases are extensive. Therapeutic strategies may involve developing small molecules or biologics to modulate 14-3-3 protein interactions with their binding partners, thus influencing key signaling pathways in cartilage integrity and inflammatory responses. Targeting these interactions could yield new treatments for OA, RA, and fractures. Additionally, 14-3-3 proteins could serve as biomarkers to monitor disease progression or treatment response, allowing for more personalized and timely interventions. Future studies should conduct large-scale clinical trials to validate the efficacy and safety of 14-3-3 targeted therapies in different patient populations. Furthermore, exploring the molecular mechanisms by which 14-3-3 proteins affect bone and joint health could identify new drug targets and intervention pathways. Finally, studying the role of 14-3-3 proteins in pain perception and their potential as analgesic targets could open new avenues for treating chronic pain associated with joint diseases. The collective data underscore the significant potential of 14-3-3 proteins as molecular targets in the diagnosis and treatment of bone- and joint-related ailments, offering the prospect of greatly improved management for these often-incapacitating conditions.
